# A Novel Combination of Accelerometry and Ecological Momentary Assessment for Post-Stroke Paretic Arm/Hand Use: Feasibility and Validity

**DOI:** 10.3390/jcm10061328

**Published:** 2021-03-23

**Authors:** Yi-An Chen, Marika Demers, Rebecca Lewthwaite, Nicolas Schweighofer, John R. Monterosso, Beth E. Fisher, Carolee Winstein

**Affiliations:** 1 Department of Occupational Therapy, Georgia State University, 140 Decatur Street, Atlanta, GA 30303, USA; 2 Division of Biokinesiology and Physical Therapy, University of Southern California, 1540 Alcazar Street, Los Angeles, CA 90089, USA; demers@pt.usc.edu (M.D.); rlewthwaite@dhs.lacounty.gov (R.L.); schweigh@usc.edu (N.S.); bfisher@usc.edu (B.E.F.); winstein@usc.edu (C.W.); 3 Rancho Los Amigos National Rehabilitation Center, 7601 E Imperial Hwy, Downey, CA 90242, USA; 4 Department of Psychology, University of Southern California, 3620 South McClintock Avenue, Los Angeles, CA 90089, USA; johnrmon@usc.edu

**Keywords:** ecological momentary assessment, accelerometry, arm/hand use, stroke

## Abstract

Use of the paretic arm and hand is a key indicator of recovery and reintegration after stroke. A sound methodology is essential to comprehensively identify the possible factors impacting daily arm/hand use behavior. We combined ecological momentary assessment (EMA), a prompt methodology capturing real-time psycho-contextual factors, with accelerometry to investigate arm/hand behavior in the natural environment. Our aims were to determine (1) feasibility and (2) measurement validity of the combined methodology. We monitored 30 right-dominant, mild-moderately motor impaired chronic stroke survivors over 5 days (6 EMA prompts/day with accelerometers on each wrist). We observed high adherence for accelerometer wearing time (80.3%), EMA prompt response (84.6%), and generally positive user feedback upon exit interview. The customized prompt schedule and the self-triggered prompt option may have improved adherence. There was no evidence of EMA response bias nor immediate measurement reactivity. An unexpected small but significant increase in paretic arm/hand use was observed over days (12–14 min), which may be the accumulated effect of prompting that provided a reminder to choose the paretic limb. Further research that uses this combined methodology is needed to develop targeted interventions that effectively change behavior and enable reintegration post-stroke.

## 1. Introduction

Daily spontaneous use of the paretic arm and hand after stroke is one of the most important recovery indicators in neurorehabilitation [1,2]. An implicit assumption behind current rehabilitation approaches is that improvements of arm/hand motor capacity will automatically translate into functional performance and use of the paretic arm/hand in the natural environment. However, there is mounting evidence that challenges this assumption [3]. One of the earliest studies reported that, although motor capability was demonstrated in the rehabilitation hospital, 52% of stroke survivors did not incorporate the paretic arm/hand when performing daily activities at home [4]. Even in patients with full motor recovery (as indicated by a maximum score of the Fugl-Meyer Upper Extremity Assessment, FMA), 27% reported limited hand use in daily activities [5].

This evidence suggests that there is a “translation gap”—a gap between what people can physically do (i.e., recovered motor capability) and what people actually do (i.e., daily arm/hand use) at home in the daily context. Although this gap was first identified over 40 years ago [4], it is still commonly observed today. Limited use of the paretic arm/hand not only leads to restricted recovery and further functional degradation, but it also constrains activity and participation [1,2]. To effectively enhance an individual’s daily paretic arm/hand use, we need to better understand the factors that may impact the translation of arm/hand capability into spontaneous use. Interventions that target those factors may effectively minimize the gap and maximize stroke survivors’ recovery, daily function, and overall well-being.

A sound methodology is a necessary first step to identify the putative factors impacting daily arm/hand use behavior. Recently, wrist-worn accelerometers have emerged as a reliable and valid tool to measure accelerations of arm/hand movement in the natural environment [6]. This technology provides an objective index of daily arm/hand use behavior after stroke [7,8,9,10,11]. However, there are critical limitations to this emerging technology [12]. For example, researchers have developed different methods, such as spectral analysis [13], pattern-recognition [9,11], and machine learning algorithms [14,15] to understand acceleration signals. Nevertheless, it is still very challenging to identify specific daily activities from acceleration signals [16]. Most importantly, although accelerometry can be used to objectively quantify day-to-day arm/hand use (e.g., movement activity counts), it lacks important contextual information about the individual’s psychological state and social environment [17]. Factors such as self-efficacy, mood, and social environment have been shown to play an essential role in functional behavior after stroke (e.g., balance, walking) [18,19,20], and are therefore likely to be critical determinants of daily paretic arm/hand behavior. For instance, an individual’s confidence level for a particular daily activity in different contexts (e.g., at home with family or in public with strangers) may affect his/her choice to use the paretic limb for that activity. Exclusive use of accelerometry would miss this kind of essential information and thereby impede a more comprehensive understanding of arm/hand use behavior after stroke.

To address these limitations, we combined accelerometry with a well-established methodology from behavioral science and behavioral medicine, ecological momentary assessment (EMA), to obtain the psychosocial and contextual information that accompanies post-stroke arm/hand use behavior. EMA is a mobile-based prompt methodology that uses brief electronic questionnaires to capture real-time self-reported behavioral and psycho-contextual variables (e.g., concurrent activities in which participants engage, self-efficacy, mood, and social contexts) [21]. Researchers have successfully used EMA to track physical activity levels in non-disabled older adults [22,23] and to monitor depressive and anxiety symptoms in the stroke population [24,25,26]. To the best of our knowledge, this is the first study to utilize EMA combined with accelerometry to gain a more complete understanding of daily paretic arm/hand behavior post-stroke.

Our first aim was to determine the feasibility of combining accelerometry with EMA to assess paretic arm/hand behavior and related psycho-contextual information in individuals with stroke in the natural environment. Participants were asked to wear accelerometers and respond to six EMA prompts per day over a five-day monitoring period. Feasibility of the combined methodology was evaluated by examining (a) accelerometer wearing time, (b) EMA response rates, and (c) participant’s feedback and acceptability of the combined methodology through an exit interview. Previous research with chronic stroke survivors reported that the average accelerometer wearing time ranged from 76% to 89% of waking hours (i.e., around 13–15 h of wearing with approximately 7 h of daily sleep/day) [7,8]. The overall EMA response rate in stroke-related studies was also reported to range from 74% to 84% [24,25,26]. We expected an acceptable engagement with the combined methodology to be at least 75% of wearing time and response rate.

Our second aim was to establish measurement validity of the combined methodology. Previous studies suggest that EMA can be subject to response bias and measurement reactivity [27,28]. *Response bias* is defined as potential response favoritism in that there may be situations for which participants are more or less likely to respond to EMA prompts with respect to the monitored behavior [27,28]. *Measurement reactivity* describes a behavior change induced simply by the act of evaluating the monitored behavior [27,28]. Individuals may assign unusually high attention to the monitored behavior and alter that behavior simply because it is being monitored. Our participants might have been expected to intentionally or unintentionally use the paretic arm/hand more frequently than usual. Therefore, before proceeding with a complete analysis of the psycho-contextual factors that influence arm/hand use behavior, we wanted to carefully examine the validity of the combined methodology by investigating (a) EMA response bias and (b) measurement reactivity.

## 2. Materials and Methods

### 2.1. Participants

Thirty participants were enrolled if they met the following inclusion criteria: (1) pre-morbidly right-hand dominant as determined by a modified Edinburgh Handedness Questionnaire [29], (2) left hemisphere stroke with right-side paresis, (3) at least minimal arm/hand motor capacity as measured by the FMA [30] (total motor score ≥ 20, sub-score of the finger mass flexion ≥ 1), (4) community-dwelling, (5) capability to read and communicate in English, and (6) capacity to learn and use the accelerometers and EMA smartphone following instruction and training. Exclusion criteria were (1) moderate to severe cognitive deficits as measured by the Montreal Cognitive Assessment (MoCA score < 16) [31], (2) unilateral spatial neglect as measured by the Albert’s Test [32], (3) pain or musculoskeletal problems in the paretic limb that affected general arm/hand use, and (4) any active medical or neurological condition that would interfere with participation. A sample size of 30 was determined based on the one-in-ten rule of thumb [33] (i.e., 10 participants per variable) for a planned regression analysis with three psycho-contextual factors (i.e., self-efficacy, mood, social environments measured with EMA) to determine the impacts of those factors on arm/hand use as part of a future project.

We exclusively recruited individuals with right, dominant-side hemiparesis (i.e., their more paretic extremity was their premorbidly dominant right arm/hand). Participants who have a non-dominant-side stroke demonstrate less paretic arm use [34], greater hand impairment [35], and less improvement after training [36] than those with a dominant-side stroke. To avoid the possible confounding effects of handedness on paretic arm/hand use in this small sample, we controlled for hand dominance and side of stroke.

Prior to enrollment, all participants read and signed an Informed Consent form according to the standard procedures of the University of Southern California (USC) Health Sciences Institutional Review Board.

### 2.2. Study Design

**Figure 1:**
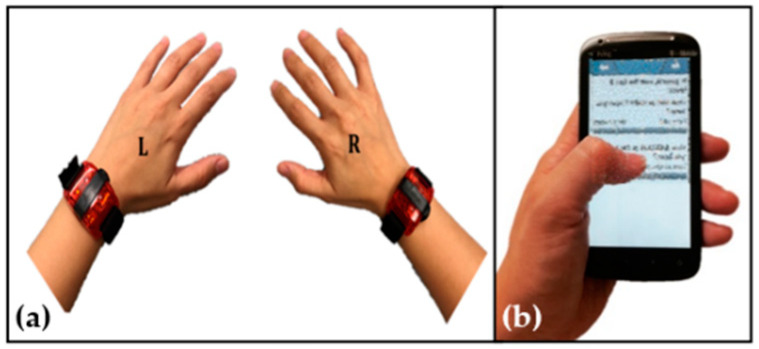
Device configuration of (**a**) accelerometers and (**b**) ecological momentary assessment (EMA) smartphone.

This was a 5-day observational study in which participants were asked to wear one accelerometer on each wrist and to respond to 6 EMA prompts per day (Figure 1a,b). Two visits to the Motor Behavior and Neurorehabilitation Laboratory on the USC Health Sciences Campus, scheduled before and after the monitoring period, were required for screening, outcome measure acquisition, and familiarization with and equipment return.

### 2.3. Instruments

The ActiGraph accelerometer with a tri-axial sensor (wGT3X-BT model; ActiGraph, Inc. Pensacola, FL, USA) was used to capture the movement acceleration of participants’ daily arm/hand behavior (30 Hz sampling rate). Each accelerometer included a Velcro strap for ease of donning/doffing at the wrist.

EMA data were collected through a mobile smartphone (HTC Sensation, AT&T USA Dallas, TX, USA) installed with custom software, movisensXS (Version 0.6.3658, movisens GmbH, Karlsruhe, Germany). This software platform allows one to program prompt schedules, display questions, save participants’ responses, and record time logs, including prompt notification, start, and completion times. The software disabled the mobile phone functions including sending and receiving calls, texting, and internet browsing. EMA prompts were time-stamped to allow synchronization with accelerometry data.

### 2.4. Procedure

During the first laboratory visit, participants were asked to complete a demographic form and a battery of screening/outcome assessments (e.g., modified Edinburgh Handedness Questionnaire, FMA, MoCA, and Albert’s test). The accelerometers and EMA smartphone were provided to participants with verbal and written operation instructions. Participants were required to demonstrate the capability to don the accelerometers and to use the EMA smartphone independently after practicing with the experimenter (Y.C.). A customized EMA schedule was negotiated with each participant to best accommodate their daily routine with prompts spaced out by at least 2 h. A study overview sheet including reminder messages (e.g., “remember to charge the phone every night”) was given to each participant before they left the lab.

During the 5-day monitoring period, participants were asked to wear the accelerometers and to carry the EMA smartphone with them during waking hours. Participants were instructed to take off the accelerometers during activities for which the Velcro straps might get wet and cause discomfort (e.g., showering, swimming), and while sleeping. Participants were prompted by an auditory signal from the smartphone, 6 times per day, to respond to EMA questions. Upon receiving an EMA prompt, participants were instructed to stop any ongoing activity, provided it was safe to do so, and respond to the EMA questions. Each EMA prompt included a total of 17 questions (see Supplementary Material S1 for details): 6 questions regarding arm/hand use behavior (e.g., whether there was any arm/hand use right before prompted) and 11 questions related to psycho-contextual information (3 for self-efficacy, 4 for mood, and 4 for physical/social context). If no response was initiated within 5 min, the phone emitted two additional reminder signals at 5-min intervals. Afterwards, the prompted EMA questions became inaccessible until the next prompt. If a prompt occurred during an incompatible activity (e.g., driving or showering), participants were instructed to ignore it. In addition to the 6 scheduled prompts/day, participants were encouraged to self-initiate an EMA prompt anytime that they desired (i.e., self-triggered prompt).

After the first day of data collection, the experimenter (Y.C.) called each participant to clarify concerns, answer questions, and/or resolve technical issues if any arose. A study contact phone number was also available to the participant to report problems at any time. Participants returned to the laboratory for a second visit after the monitoring period to return all devices and to complete an exit interview.

### 2.5. Data Analysis—Accelerometry

Raw accelerometry data (m/s^2^) were downloaded from the sensors and processed using ActiLife 6.0 software (ActiGraph, Inc. Pensacola, FL, USA) with the methods commonly used in the field [7,8,9,10,11]. The data from each axis (x, y, z) were filtered using a band-pass filter (0.25–2.5 Hz) and binned into 2-s epochs. Within each epoch, the acceleration values of each sampling point were summed together (i.e., 30 Hz sampling rate × 2 s = 60 samples/epoch) and converted into an “activity count” value using the manufacturer’s proprietary algorithm (e.g., 1 activity count = 0.01664 g for an acceleration produced by a movement with a frequency of 0.75 Hz) [37].

The activity count from each axis was further combined into a single resultant value (=√x^2^ + y^2^ + z^2^) for each epoch, using a custom-written MATLAB program (version R2015a) (The MathWorks Inc., Natick, MA, USA). A threshold of 2 (in activity count), validated by others [7,8,9,10,11] and by our own pilot testing, was then used to define participants’ daily arm/hand movements using the resultant activity count. When the activity count was ≥2 in an epoch, the arm/hand was defined as moving (“movement”) during that 2-s epoch period. When the activity count was <2, the arm/hand was defined as not moving (“no movement”).

The “movement” accelerometry signal was further categorized into unimanual and bimanual arm/hand movements. Unimanual right (R) or left (L) arm/hand movement was defined when the activity count from one but not two accelerometers was ≥2 during the epoch (i.e., only one arm/hand was considered moving at that time). Bimanual arm/hand movement was defined when the activity counts from both right and left accelerometers were ≥2 (i.e., both arms/hands were considered moving during the epoch). Time duration in seconds of each “movement” epoch was then summed (each epoch was 2 s) for either unimanual R (paretic), unimanual L, or bimanual (B) arm/hand movements (TimeR, TimeL, and TimeB). These movement time metrics were the variables of interest and the indices we used to quantify the amount of arm/hand use during the 5-day monitoring period.

**Figure A1:**
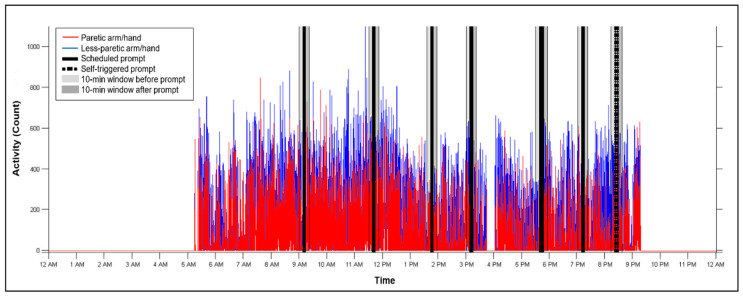
Synchronization of accelerometer data with EMA prompt times. An example from one participant for 24-h accelerometry data.

Using the time stamp, we then synchronized the EMA prompts with the accelerometry data and delineated a 10-min window of accelerometry data *right before* participants started each prompt and *right after* they completed the prompt (Figure A1) for validity testing (Aim 2). We based the 10 min on previous literature that examined physical activity levels in older adults [38,39]. Our pilot testing also found no systematic difference among different window lengths of 3, 5, 10, and 15 min.

### 2.6. Outcome Measures

#### 2.6.1. Aim 1: Feasibility

- *Accelerometer wearing time*: We calculated each participants’ overall and daily accelerometer wearing time to rule out novelty or fatigue effects during the 5 days. If the participant showed “no movement” for any 3-h segment (i.e., the activity counts in every epoch within the 3-h period were <2), we considered that as non-wearing time [8]. Accelerometry data were included in further analyses only when there was a clear indication that both accelerometers were worn.
- *EMA response rates*: Participants received a total of 30 scheduled prompts (6×/day for 5 days) and could self-trigger a prompt anytime during participation. Duplicate prompts were defined when (1) the time interval between 2 adjacent prompts was less than 1 min and (2) the responses of the 2 prompts were the same on the close-ended EMA questions. After removing duplicate prompts, the overall response rate and the response rate by day were calculated for each participant and then averaged across participants for both scheduled and self-triggered prompts.
- *Participants’ feedback*: During the exit interview, participants were asked whether they experienced any problems or technical issues with either device (open-ended questions). Their responses were noted and summarized by the experimenter (Y.C.). Specific to EMA, we asked three 7-point Likert-type questions: (a) “To what extent, did you find responding to the survey questions easy to do?” (1 = not easy at all; 4 = neutral; 7 = very easy), (b) “Generally, what do you think of the total number of prompts you got?” (1 = too many prompts; 4 = about right; 7 = need more prompts to reflect the activities I did), and (c) “To what extent, did you find the prompts on the smartphone disruptive in your day?” (1 = not disruptive at all; 4 = neutral; 7 = very disruptive). Participants were also encouraged to share feedback or comments, such as the reasons for missing prompts and their use of the self-trigger function.

#### 2.6.2. Aim 2: Validity

- *EMA response bias*: We evaluated whether the likelihood of responding to EMA prompts was related to participants’ arm/hand use behavior. We compared the time metrics (i.e., TimeR, TimeL, and TimeB) within the before-prompt 10-min window between answered and unanswered scheduled prompts. If, for example, no response to prompts was associated with lower levels of arm/hand movement, it would suggest that participants might have dismissed the EMA prompt due to social desirability to avoid answering “no use” when they did not use their arm/hand. Similarly, the time metrics within the before-prompt 10-min window were also compared between the answered scheduled prompts and the self-triggered prompts to evaluate whether participants tended to have higher arm/hand use levels prior to a self-triggered EMA prompt.
- *EMA measurement reactivity*: We examined the immediate and accumulative effects of EMA measurement reactivity. To determine the immediate effect, we compared the arm/hand movements before and after each EMA prompt. A significant difference of the time metrics between before- and after-prompts would suggest that the act of monitoring behavior itself may have influenced arm/hand use behavior. The accumulative effect was examined by comparing the time metrics of each day across the 5 days. To facilitate comparison across participants, the daily time metrics were normalized by each participant’s daily wearing time and converted to a percentage.

Note that we did not include a comparison of arm/hand use between the objective accelerometry measure and the subjective EMA arm/hand use report in this study because it is beyond the scope of our aims.

### 2.7. Statistical Analysis

Repeated measures analysis of variance (ANOVA) with post-hoc Bonferroni correction was conducted to examine whether participants’ accelerometer wearing time, EMA response rates, and arm/hand movement (EMA measurement reactivity of accumulative effect) changed over days. Day was set as the within-subject factor with 5 levels (Day 1 to 5).

Hierarchical linear regression modeling (HLM) was used to examine EMA response bias and the immediate effect of measurement reactivity (before vs. after prompts). Compared to the repeated measures ANOVA, HLM allows different numbers of measurements (unequal cell sizes) when accounting for the variances in each level (e.g., different total numbers of EMA prompts completed among participants, or different numbers of EMA prompts completed among days for each individual). To accommodate the multilevel structure of our data, a two-level HLM (between-participant and within-participant levels, i.e., using participants as random intercepts) was used for each time variable to examine the changes of arm/hand use as a function of prompt type (answered vs. unanswered, scheduled vs. self-triggered) and before-/after-prompt for EMA response bias and immediate effect of measurement reactivity, respectively.

All statistical analyses were performed using Stata 14.2 (StataCorp, College Station, TX, USA). Data were log transformed if not normally distributed.

## 3. Results

### 3.1. Participants

Participants were on average 61.2 years old, 4.7 years from stroke onset, with an average FMA score of 47.3 out of 66. Motor impairment was classified as mild for 19 participants, moderate for 6, and severe for 5 [40]. Characteristics of each participant are provided in Table 1.

### 3.2. Aim 1: Feasibility

#### 3.2.1. Accelerometer Wearing Time

The average daily accelerometer wearing time across the five days was 13.7 ± 0.7 h (approximately 80.3% of waking hours). Participants showed a lower accelerometer wearing across successive days (~0.4 h less each day, Table 2). However, no significant difference was found in wearing time across days based on repeated measures ANOVA (F_4, 29_ = 1.38, *p* = 0.259). The wearing time for all five days remained above 75% of waking hours.

#### 3.2.2. EMA Response Rates

**Figure 2:**
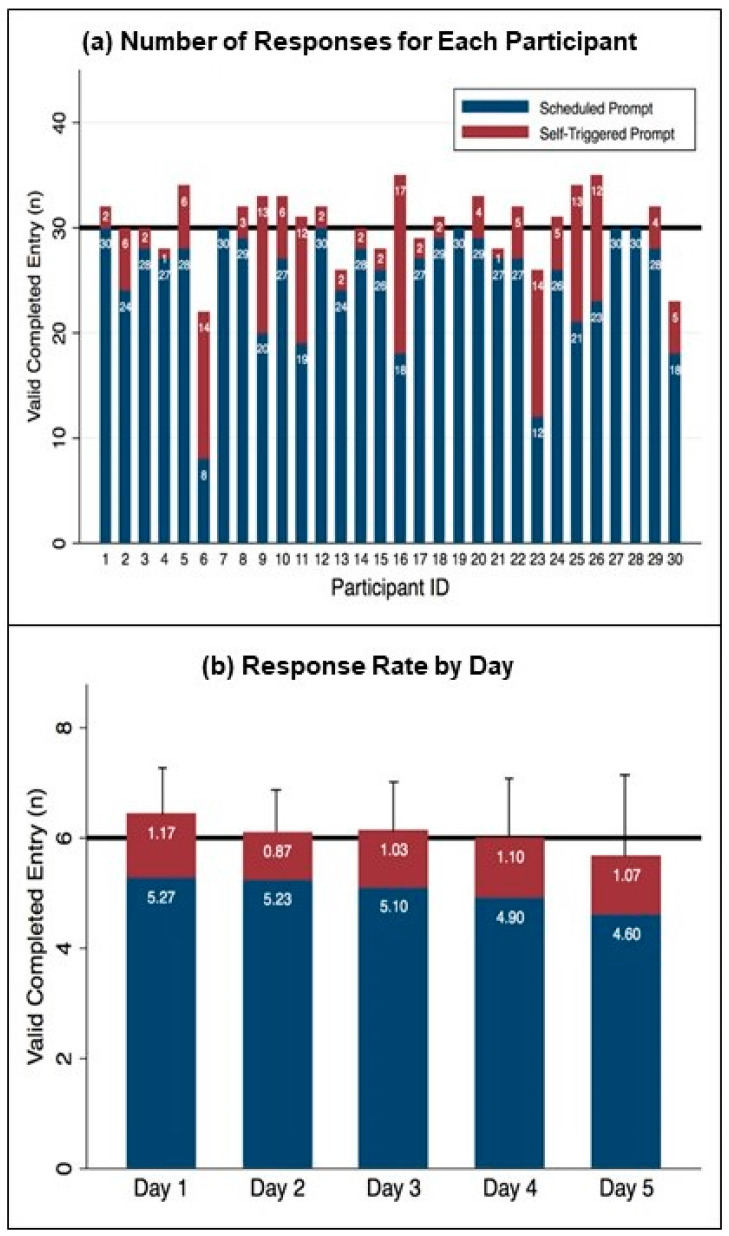
High feasibility of EMA response rate for scheduled (Blue) and self-triggered (Red) prompts. (**a**) Number of responses for each participant. The goal of 30 prompts during participation (6 prompts/day for 5 days) is indicated by the black horizontal line. (**b**) Response rate by day. The goal of 6 prompts per day is indicated by the black horizontal line. The error bars represent the standard deviation of each bar.

Of the total 900 scheduled EMA prompts (30 prompts × 30 participants), 10 prompts (1.1%) were not provided due to suspected technical problems (6 prompts) or the phone being accidentally turned off (4 prompts from one day of a single participant). A total of 754 out of the 890 provided prompts were completed. The average overall response rate was 84.6 ± 18.5% across participants, which represented an average of 25.1 ± 5.6 completed prompts out of the 30 scheduled prompts. Each participant’s number of responses is shown in Figure 2a (blue bars).

After removing duplicate prompts, participants self-triggered a total of 157 valid prompts during the five -day monitoring period. The majority of participants (*n* = 26) self-triggered at least 1 prompt and up to 17 prompts with an average of 5.2 ± 5.1 additional prompts (Figure 2a, red bars). There was a strong negative correlation between the number of completed/answered scheduled prompts and self-triggered prompts (Spearman *r* = −0.721, *p* < 0.001), suggesting that participants initiated fewer prompts when they completed more scheduled prompts.

Overall, participants completed an average of 30.3 ± 3.1 prompts (range, 22–35 prompts) over five days, including both scheduled and self-triggered prompts. The average time to complete a single EMA prompt was 4.0 ± 1.7 min across participants. EMA response rate trended down by day (Figure 2b); however, there was no significant effect of day for either the scheduled or self-triggered prompt response rate (F_4, 29_ = 2.52, *p* = 0.092; F_4, 25_ = 0.08, *p* = 0.877, respectively).

#### 3.2.3. Participants’ Feedback

Participants did not report any major technical issues using the accelerometers. Five participants mentioned that the accelerometer sensors were bulky, but a majority (*n* = 26) perceived that donning and doffing was easy.

All participants reported that responding to EMA questions was straightforward and easy (average, 6.6 ± 0.6; range, 5–7; 7 = very easy). The total number of prompts was also well accepted for the majority (*n* = 28; 4.4 ± 1.2), who rated four (about right) or above (need more prompts). Participants perceived that responding to EMA prompts was not disruptive in their daily life (3.0 ± 1.7; 1 = not disruptive at all, 4 = neutral).

Participants also shared that the common reasons that they missed the scheduled prompts were (a) they were in the middle of an activity that could not be interrupted (*n* = 10), such as driving or at work, (b) they preferred not to be interrupted (*n* = 6), such as while exercising or at a football game, (c) they decided not to bring the EMA phone with them due to the possible damage to it during the activity (*n* = 4), such as showering or swimming, and (d) they forgot to bring the phone with them at that time (*n* = 4).

In addition, over one-third of the participants (*n* = 11) mentioned that they triggered a prompt when they knew that they had missed or would have missed a scheduled prompt. The overall feedback on the self-trigger function of EMA was positive, for example:

- “The [self-triggered] function is a great idea. I know I can make up some … so I didn’t feel too worried when I missed a survey.”
- “I think it is great. I can make sure that I did at least 30 prompts during the week.”
- “It is really hard to follow the [prompt] schedule for an active person like me. … I usually enter one [prompt] when I have time.”

### 3.3. Aim 2: Validity

**Figure A2:**
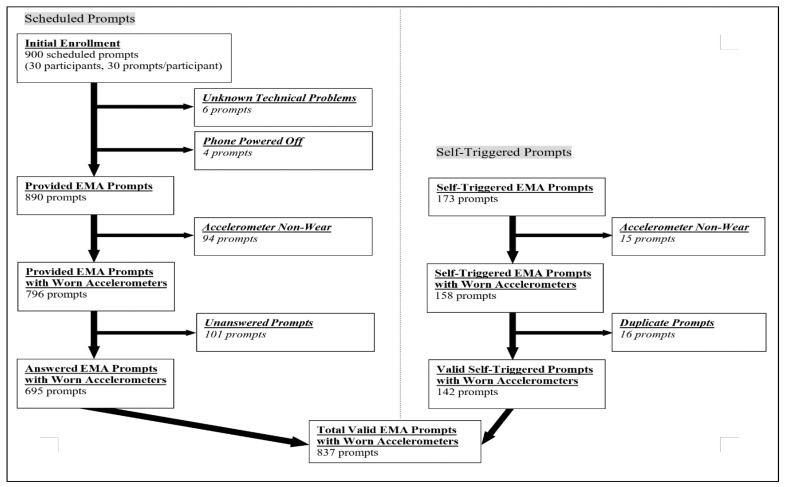
Flow chart of data availability.

After synchronizing the EMA prompts with the accelerometry data, a total of 837 valid EMA prompts (both scheduled and self-triggered) and accelerometer pairings were included in the following validity analyses. A flow chart (Figure A2) illustrates data availability and sources of missing data.

#### 3.3.1. EMA Response Bias

We found no significant difference for the time metrics between answered and unanswered prompts prior to the EMA prompt (TimeR, *p* = 0.562; TimeL, *p* = 0.220; and TimeB, *p* = 0.130). Likewise, the time metrics did not differ between the scheduled prompts and the self-triggered prompts (TimeR, *p* = 0.130; TimeL, *p* = 0.809; and TimeB, *p* = 0.066). Therefore, we did not find evidence that participants responded to the EMA prompts on the basis of the prior amount of arm/hand use as measured by our time metrics.

#### 3.3.2. EMA Measurement Reactivity

We observed no significant change in the time metrics before vs. after answering an EMA prompt (TimeR, *p* = 0.275; TimeL, *p* = 0.169; and TimeB, *p* = 0. 293). Therefore, there was no *immediate* effect of EMA measurement reactivity on arm/hand movement.

**Figure 3:**
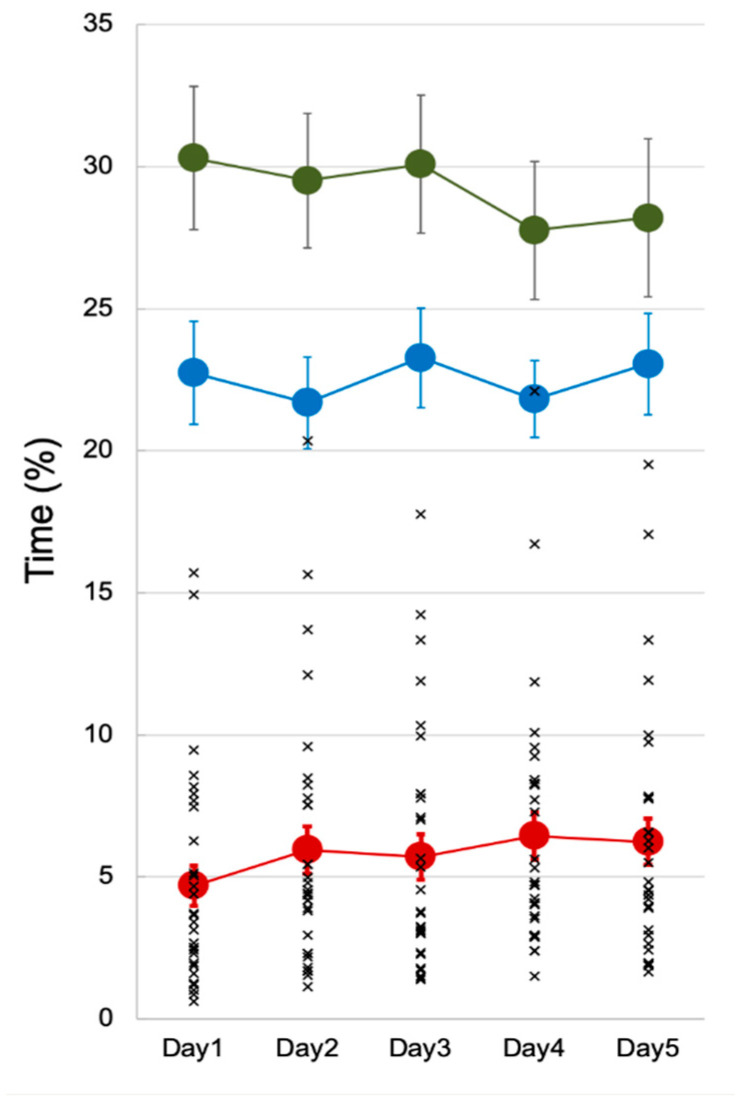
EMA accumulative measurement reactivity. The solid color circles represent the daily average of the time metrics in percentage. Error bars represent standard errors. The cross (x) represents each individual’s TimeR for each day to show a uniform trend of increase observed in the participants. Abbreviations: TimeR = time duration of the unimanual right (paretic) arm/hand movement; TimeL = time duration of the unimanual left arm/hand movement; TimeB = time duration of the bilateral arms/hands movement.

However, there was evidence of an *accumulative* effect of EMA measurement reactivity over days. We observed a small, but nevertheless, significant increase in unimanual right paretic arm/hand movement over days (*p* < 0.05; TimeR, Table 2 and Figure 3). Post-hoc comparisons demonstrated a significant increase in time (%) in the unimanual right paretic arm/hand (TimeR, Table 2) from Day 2 to Day 5 compared to Day 1 (*p* < 0.001 ~*p* = 0.002), and for Day 4 compared to Day 3 (*p* = 0.045). For interpretation and discussion purposes, we converted percent time back to actual time units (min or hour) using the group daily average accelerometer wearing time (i.e., 13.7 h wearing/day over 5 days, Table 2). Participants’ average time using their paretic arm/hand significantly increased from 38.5 min on Day 1 to the highest of 52.7 min on Day 4. Bimanual activity over days (TimeB, Table 2 and Figure 3) revealed a decreasing trend; however, this trend did not reach significance (*p* = 0.197). Similarly, there was no significant change in the use of the non-paretic left limb over days (*p* = 0.578; TimeL, Table 2 and Figure 3).

## 4. Discussion

To obtain a more comprehensive understanding of stroke survivors’ daily arm/hand use behavior, we used a novel assessment that combined accelerometry with EMA to capture paretic arm/hand use in the natural environment. Here, we discuss the feasibility and validity of the combined methodology. Our findings suggest that the combined methodology is a feasible tool with high use of both accelerometers (average wearing time ~80% of waking hours) and EMA (average response rate ~85%) and generally positive user feedback in our chronic stroke survivor cohort. Further, the absence of response bias and immediate effect of EMA measurement reactivity indicate good measurement validity of the combined methodology. However, we observed a small but significant cumulative effect that manifested as an increase in unimanual paretic (right) arm/hand movement over days. The incremental arm/hand movement observed only for the paretic side deserves further discussion.

### 4.1. High Feasibility of the Combined Methodology

Our feasibility findings are consistent with other previous studies reporting accelerometer wearing time [7,8] and EMA response rate [24,25,26] in stroke survivors. With our unique five-day design, we further demonstrated that there was no significant decrease over days in use of the combined methods. To the best of our knowledge, no other stroke studies have reported daily feasibility data for either accelerometer wearing time or EMA response rate. Our results, including participant feedback, suggest that the combined methodology is acceptable to chronic stroke survivors for at least a five-day period.

### 4.2. The Advantages of EMA Self-Triggered Function

This was the first time that the self-trigger function of EMA was employed in the stroke population. Our original purpose for implementing this function was to increase the potential to capture arm/hand behavior. Since arm/hand movements are single discrete events that occur in the daily context, rather than constant or sustained conditions of mind (such as depression and anxiety), we reasoned that the self-trigger function might be of benefit to capture these more discrete arm/hand behavior events, especially in the stroke population in which use of the paretic arm/hand is often limited.

Based on participants’ responses in the exit interview, we found that the self-trigger function was primarily used to compensate for missed prompts rather than to enhance arm/hand use counts. The negative correlation between the answered scheduled prompts and the self-triggered prompts confirms and quantifies the compensatory role of the self-trigger function. Participants’ positive feedback about the self-trigger function further revealed that they felt less nervous when they missed prompts. This function also provided flexibility to people who had difficulty following the prompt schedule due to a busy life. Participants were able to meet use expectations without overly disrupting their daily activities. The high use of the combined methodology likely reflects the benefits of autonomy support provided by the self-trigger option. In this study in which direct clinical benefit was not expected, participants with stroke in effect collaborated with the investigators to generate insights about their paretic arm/hand use, making efforts to provide sufficient data for that knowledge. A degree of comfort with less worry was provided when they knew they could ignore the scheduled prompt and complete it when "they" chose to do so.

It is worth noting, however, that unlike the scheduled prompts, self-initiated responses carry the prospect of self-selection effects. Participants were probed by the experimenters when responding to the scheduled prompts but actively responded to their own impositions for the self-triggered prompts. To avoid potential confounds of this natural difference, we carefully examined our data and conducted separate analyses of the scheduled and the self-triggered prompts. Importantly, we did not find any significant differences in responses between the two types of prompts in determining the feasibility and validity of EMA.

### 4.3. No EMA Response Bias Associated with Arm/Hand Movement

Our findings of no immediate response bias indicated that the act of EMA responding was not associated with the just-prior arm/hand use behavior. Participants did not respond, avoid, or self-trigger the EMA prompts based on whether they used or did not use their paretic arm/hand before the prompt. This finding aligned well with the exit interview data showing that participants commonly missed prompts due to an incompatible activity (e.g., showering), and that they self-triggered a prompt as a way to compensate for a missed prompt.

### 4.4. Unexpected Accumulative EMA Measurement Reactivity: Increased Right (Paretic) Arm/Hand Movement over Days

A previous study [22] showed an immediate effect of EMA measurement reactivity on sedentary activity in non-disabled adults. Individuals who were overweight and obese significantly increased sedentary activity after completing an EMA prompt [22]. On the contrary, while our participants did not show an immediate change of arm/hand behavior after responding to EMA, we did observe a small but significant increase in right (paretic) arm/hand movement over days. Given that our study was designed to monitor but not intervene in arm/hand use, this unexpected increase deserves more discussion.

EMA has been considered “a part of behavior-change treatment, not just an assessment” [28]. Evidence supports that EMA may function as “treatment” (i.e., ecological momentary intervention [EMI]) [27,28], possibly due to the extra attention that is drawn to the monitored behavior. As such, the extra attention may have provided the impetus for the small but significant accumulated increase in paretic arm/hand movement that we observed. Some participants commented that, when responding to the EMA arm/hand questions, they realized that they had not used the paretic arm/hand as much as they should have given their motor capability (e.g., “I became so aware that I haven’t been using my right [paretic] hand much... I should have tried to do more things with my [paretic] hand. …”). Through responding to the arm/hand use question, we speculate that EMA may have acted as EMI providing a kind of reminder and in so doing, altered participants’ mindset. The consistent daily prompts may influence participants’ behavior toward more use over time [41]. This suggests the need for future investigations of EMI to enhance the use of the paretic arm/hand in the natural environment.

Although this accumulated increase in paretic limb movement time was statistically significant, it is not clear whether a 12–14 min increase (Day 4 and 5 compared to Day 1) over an average of 13.7-h daily monitoring period (i.e., the average accelerometer wearing/day) is indicative of a meaningful or functional arm/hand activity increase. Further examination is needed to understand the meaningfulness of this change. The 12–14 min increase was around one-third of paretic arm/hand use on Day 1 (38.5 min; Table 2 and Figure 3). This increase was also a trend observed in 77% of our sample (23/30 participants). Additional analysis excluding the inactivity time (i.e., no movement from either hand) showed the same significant increase in the right arm/hand use over days (*p* < 0.001). Thus, our results support the interpretation that, when participants performed daily activities that required arm/hand use, they indeed increased the use of the paretic hand over the five days.

Alternatively, the significant daily increase of right arm/hand movement may have occurred simply due to abnormally low activity on Day 1, possibly caused by observation-induced anxiety or device unfamiliarity, etc. To compare the data from previous studies, we calculated the data reported by Bailey and colleagues [10] and found a 3.3% paretic arm/hand movement time during a 24-h wearing period (=0.8 h of paretic arm/hand movement/24 h × 100%). This suggests that our 4.7% (Table 2 and Figure 3) on Day 1 was not abnormally low and may be well within an expected range.

### 4.5. Limitations

We have identified four principal limitations to this study. First, we did not include a control group, as our aims were to determine the feasibility and validity of the combined methodology to assess paretic arm/hand behavior after stroke. However, the addition of a control group of stroke survivors who wear the accelerometers without using EMA would allow us to better deconstruct the cumulative effect of measurement reactivity, as well as refine alternative explanations (e.g., apparent low activity on Day 1). Second, although all the participants demonstrated that they were able to independently use the EMA smartphone during Visit 1, we could not rule out the possibility of caregiver assistance for EMA responding and the potential influence on responses from caregivers. Third, the accelerometry data only provides a proxy measure of goal-directed arm/hand use behavior. Fine motor activities, especially involving dexterous finger movements, may be missed, because the sensors were worn on the wrists and are not sensitive to the small movement of the fingers. Non-purposeful movements, such as arm swing during walking, however, may be recorded and are difficult to distinguish from goal-directed arm/hand movements without using an additional sensor worn on the hip or lower extremity. Lastly, in order to limit variance in this small sample, we only recruited participants with left-hemisphere (dominant-hand affected) stroke, which limits the generalizability of our findings to other stroke populations. Future investigations with a larger sample should include those with right-hemisphere stroke and examine hemisphere-specific arm/hand use behavior that will more inclusively inform the potential factors likely to impact the translation gap in stroke recovery.

## 5. Conclusions

We demonstrated the feasibility of a novel methodology, which combined accelerometry with EMA, and showed promise for advancing understanding of post-stroke arm/hand use behavior in the daily environment. The validity testing also confirmed that the arm/hand use behavior did not immediately affect the EMA response nor vice versa. These findings support the use of the combined methodology to measure arm/hand movement and related psycho-contextual factors potentially impacting daily paretic arm/hand use behavior after stroke. Future work from our group will examine the data collected from the combined methodology to identify the key factors that influence decisions to use the paretic arm/hand with the long-term goal to minimize the translation gap in individuals with chronic stroke. The unexpected increase of paretic limb movement time over days may have resulted from an accumulated effect of measurement reactivity, whereby EMA functioned as EMI (intervention) to draw attention and implicitly remind participants to engage the paretic arm/hand. Future research is needed to further understand this small but significant increase and explore the application of EMI to increase functional paretic arm/hand use.

## Figures and Tables

**Table 1:** Characteristics of Participants (*n* = 30).

ID	Gender	Age (Years)	Onset (Years)	MoCA	FMA	
1	Male	65.3	8.1	26	61	Mild
2	Male	59.1	12.0	25	38	Moderate
3	Male	59.8	2.8	25	59	Mild
4	Female	63.6	9.0	19	30	Moderate
5	Male	31.8	1.5	21	66	Mild
6	Male	59.9	0.7	30	65	Mild
7	Male	61.2	4.6	25	58	Mild
8	Male	66.8	14.8	19	22	Severe
9	Male	60.0	1.2	25	63	Mild
10	Male	70.5	2.3	25	21	Severe
11	Female	24.4	1.7	29	48	Mild
12	Male	77.6	4.8	24	39	Moderate
13	Male	44.0	2.8	23	61	Mild
14	Male	72.3	1.9	23	66	Mild
15	Male	58.0	8.6	27	53	Mild
16	Male	75.6	5.9	20	53	Mild
17	Male	54.5	6.0	26	54	Mild
18	Male	61.7	10.6	26	52	Mild
19	Female	70.5	11.7	27	47	Mild
20	Male	79.4	2.1	23	57	Mild
21	Male	47.6	3.1	28	45	Mild
22	Female	57.6	2.8	27	55	Mild
23	Female	71.1	1.1	23	30	Moderate
24	Female	58.6	0.8	29	41	Moderate
25	Female	60.2	0.9	30	20	Severe
26	Female	73.0	3.8	19	23	Severe
27	Male	53.1	2.8	27	28	Severe
28	Female	68.7	2.8	21	40	Moderate
29	Male	82.9	1.7	30	62	Mild
30	Male	47.1	7.9	20	61	Mild
Mean ± SD	21M:9F	61.2 ± 13.1	4.7 ± 3.9	24.7 ± 3.4	47.3 ± 14.9	
(Range)	-	(24.4–82.9)	(0.7–14.8)	(19–30)	(20–66)	

MoCA = Montreal Cognitive Assessment, FMA = Upper Extremity Fugl-Meyer Assessment, M = male, F = female.

**Table 2:** Accelerometer wearing time and EMA accumulative measurement reactivity (time metrics for each day).

Variables (Mean ± SD)	Day 1	Day 2	Day 3	Day 4	Day 5
Wearing time (hours)	14.6 ± 3.2	14.0 ± 3.6	13.5 ± 3.1	13.2 ± 3.4	12.9 ± 4.3
Time (%) ^#^	-	-	-	-	-
TimeR	4.7 ± 3.9 *	6.0 ± 4.6	5.7 ± 4.3 **	6.4 ± 4.5	6.2 ± 4.5
	(38.5 min) ^&^	(48.7 min)	(46.8 min)	(52.7 min)	(50.9 min)
TimeL	22.7 ± 9.9	21.7 ± 8.8	23.3 ± 9.6	21.8 ± 7.4	23.1 ± 9.8
	(3.1 h)	(3.0 h)	(3.2 h)	(3.0 h)	(3.2 h)
TimeB	30.3 ± 13.8	29.5 ± 13.0	30.1 ± 13.3	27.8 ± 13.3	28.2 ± 15.2
	(4.1 h)	(4.0 h)	(4.1 h)	(3.8 h)	(3.9 h)

^#^ Due to positive skewness, the time (%) was log transformed before conducting the repeated measures ANOVA. We reported original percentage values here for interpretation and comparison convenience. * Bonferroni post-hoc correction: Day1 < Day2 (*p* = 0.001), Day3 (*p* = 0.002), Day4 (*p* < 0.001), and Day5 (*p* < 0.001). ** Bonferroni post-hoc correction: Day3 < Day4 (*p* = 0.045). ^&^ For interpretation and discussion convenience, time percentages were converted back to time units (min or hour) using the average accelerometer wearing time (13.7 h wearing over the 5 days). Abbreviations: TimeR = time duration of the unimanual right (paretic) arm/hand movement; TimeL = time duration of the unimanual left arm/hand movement; TimeB = time duration of the bilateral arms/hands movement.

## Data Availability

The data presented in this study are available on request from the corresponding author.

## Supplementary Materials

The following are available online at https://www.mdpi.com/2077-0383/10/6/1328/s1. Supplementary Material S1: EMA questions.
