# Supplementary material for: A Novel Combination of Accelerometry and Ecological Momentary Assessment for Post-Stroke Paretic Arm/Hand Use: Feasibility and Validity
Source: J Clin Med. 2021 Mar 23;10(6):1328. doi: 10.3390/jcm10061328 (PMC8005066; doi:10.3390/jcm10061328)
Supplement: Supplementary file 1 [file jcm-10-01328-s001.pdf]

Journal of
Clinical Medicine

MDPI

Supplementary Materials

EMA questions

For questions 1-4, 8, 13, & 15-17, the participant uses a slider bar on the EMA smartphone to indicate the point along the response range that best represents their preference level.

In general, over the last 2 hours:

1. How SAD or HAPPY have you been?
   Very sad ——————————— Very happy
2. How ANXIOUS or CALM have you been?
   Very anxious ——————————— Very calm
3. How FRUSTRATED have you been?
   Not frustrated at all ——————————— Very frustrated
4. How much ENJOYMENT have you had?
   Little enjoyment ——————————— A great deal of enjoyment
5. WHERE were you just before the phone rang?
   - Home (indoors) (laundry room, kitchen, bathroom, etc.)
   - Home (outdoors) (yard, deck, driveway, etc.)
   - Work/Volunteer work places
   - Community (park, mall, bank, restaurant, etc.)
   - Health facility/Clinic (rehabilitation therapy, etc.)
   - Vehicle (car, train, etc.)
   - Other
6. Were you ALONE just before the phone rang?
   - Yes
   - No
7. WHOM were you with just before the phone rang? (Check all that apply)
   - Family
   - Friends
   - Colleagues/Co-workers
   - Service providers (therapist, cashier, gardener, waiter…)
   - People I don't know/Strangers
   - Other
8. How would you describe your social interaction with the one(s) you were with?
   Very stressful ——————————— Very positive/uplifting
9. Please briefly describe what activity you were doing just before the phone rang.
   Text box
10. Which arm/hand(s) were you using for the activity?
    - Right arm/hand
    - Left arm/hand
    - Both arms/hands

- Neither or none

11. Which of the following(s) can describe the activity you were doing? (Check all that apply)
    - Activity that requires a lot of arm/hand STRENGTH/ENDURANCE
    - Activity that requires a high level of hand DEXTERITY/PRECISION
    - Activity that needs to COORDINATE both hands working together to accomplish
    - Activity that you were under a TIME-PRESSURE to complete
    - Activity with a NEGATIVE consequence if you failed
    - Activity with a POSITIVE consequence if you succeeded
    - None of above
12. Around how long had you been doing the activity?
    - Less than 5 min
    - 5 – 15 min
    - 15 – 30 min
    - 30 – 60 min
    - Longer than 60 min
13. How DIFFICULT was the activity to you?

    Not difficult at all ———————————————— Very difficult
14. What was your body position when the phone rang?
    - Dynamic (walking, moving around, etc.)
    - Standing
    - Sitting
    - Lying down
15. How CONFIDENT are you right now that you can use your RIGHT arm/hand to accomplish any activity that comes up or that you need to do within the next 2 hours?

    Not confident at all——————————————Very confident
16. How CONFIDENT are you right now that you can use your LEFT arm/hand to accomplish any activity that comes up or that you need to do within the next 2 hours?

    Not confident at all——————————————Very confident
17. How CONFIDENT are you right now that you can use BOTH arms/hands to accomplish any activity that comes up or that you need to do within the next 2 hours?

    Not confident at all——————————————Very confident
